# A wide range of missing imputation approaches in longitudinal data: a simulation study and real data analysis

**DOI:** 10.1186/s12874-023-01968-8

**Published:** 2023-07-06

**Authors:** Mina Jahangiri, Anoshirvan Kazemnejad, Keith S. Goldfeld, Maryam S. Daneshpour, Shayan Mostafaei, Davood Khalili, Mohammad Reza Moghadas, Mahdi Akbarzadeh

**Affiliations:** 1 Department of Biostatistics, Faculty of Medical Sciences, Tarbiat Modares University, Tehran, Iran; 2 Division of Biostatistics, Department of Population Health, NYU Grossman School of Medicine, New York, NY USA; 3 Cellular and Molecular Endocrine Research Center, Research Institute for Endocrine Sciences, Shahid Beheshti University of Medical Sciences, Tehran, Iran; 4 Department of Medical Epidemiology and Biostatistics, Karolinska Institute, Stockholm, Sweden; 5 Prevention of Metabolic Disorders Research Center, Research Institute for Endocrine Sciences, Shahid Beheshti University of Medical Sciences, Tehran, Iran

**Keywords:** Single imputation, Multiple imputations, Missing longitudinal data, Longitudinal regression tree

## Abstract

**Background:**

Missing data is a pervasive problem in longitudinal data analysis. Several single-imputation (SI) and multiple-imputation (MI) approaches have been proposed to address this issue. In this study, for the first time, the function of the longitudinal regression tree algorithm as a non-parametric method after imputing missing data using SI and MI was investigated using simulated and real data.

**Method:**

Using different simulation scenarios derived from a real data set, we compared the performance of cross, trajectory mean, interpolation, copy-mean, and MI methods (27 approaches) to impute missing longitudinal data using parametric and non-parametric longitudinal models and the performance of the methods was assessed in real data. The real data included 3,645 participants older than 18 years within six waves obtained from the longitudinal Tehran cardiometabolic genetic study (TCGS). The data modeling was conducted using systolic and diastolic blood pressure (SBP/DBP) as the outcome variables and included predictor variables such as age, gender, and BMI. The efficiency of imputation approaches was compared using mean squared error (MSE), root-mean-squared error (RMSE), median absolute deviation (MAD), deviance, and Akaike information criteria (AIC).

**Results:**

The longitudinal regression tree algorithm outperformed based on the criteria such as MSE, RMSE, and MAD than the linear mixed-effects model (LMM) for analyzing the TCGS and simulated data using the missing at random (MAR) mechanism. Overall, based on fitting the non-parametric model, the performance of the 27 imputation approaches was nearly similar. However, the SI traj-mean method improved performance compared with other imputation approaches.

**Conclusion:**

Both SI and MI approaches performed better using the longitudinal regression tree algorithm compared with the parametric longitudinal models. Based on the results from both the real and simulated data, we recommend that researchers use the traj-mean method for imputing missing values of longitudinal data. Choosing the imputation method with the best performance is widely dependent on the models of interest and the data structure.

**Supplementary Information:**

The online version contains supplementary material available at 10.1186/s12874-023-01968-8.

## Background

Longitudinal data collected from the same subjects over time are frequently used in observational studies and clinical trials. Traditional models for longitudinal data analysis are generalized linear mixed-effects models (LMM), marginal models like generalized estimating equations (GEE), and transitional models. In various types of studies, especially longitudinal ones, researchers frequently face significant challenges, such as missing data. During follow-up, some subjects may withdraw or become lost to follow-up at planned visits. Subjects who participate only during a particular study period may complete only a subset of the information [1, 2].

Most conventional statistical models deal only with complete cases, and missing data are omitted before fitting statistical models (this is the default in most statistical software programs and is called the listwise deletion method). Excluding these observations has disadvantages, including loss of information, loss of precision, reduction in statistical power, and potentially biased estimates [3]. Therefore, different approaches have been introduced to impute missing values and can be classified as either single-imputation (SI) or multiple-imputation (MI).

MI methods for imputing missing data in software programs are based on two approaches: joint modeling (JM) and fully conditional specification (FCS). JM approaches for MI are based on the multivariate distribution or the joint distribution of incomplete variables (often, the multivariate normal (MVN) distribution is considered and can be referred to as multivariate normal imputation (MVNI)) [4]. In FCS approaches, missing observations of each incomplete variable are imputed given all the other predictor variables, cycling iteratively through a sequence of univariate imputation models [5].

Several JM and FCS methods, like JM-MVN (joint multivariate normal imputation) and FCS-standard approaches, have been proposed to handle missing values in cross-sectional studies [6–11]. These approaches are also appropriate for imputing missing values in balanced longitudinal data where longitudinal measurements are obtained at fixed time intervals. In this case, the JM-MNV and FCS-standard methods treat time-dependent variables as distinct variables (wide format) for the imputation of balanced missing longitudinal data [3, 5].

Sometimes, longitudinal data are collected at unequal time intervals along with many longitudinal predictor variables. Then standard JM-MVN and FCS methods cannot be used for imputing missing values in this case because large numbers of time-dependent predictor variables may lead to problems like overfitting and multicollinearity among distinct predictor variables.

However, ignoring the longitudinal and multilevel structures when imputing missing values of longitudinal data and multilevel data may lead to biased inferences for the estimates of regression coefficients and their standard errors [12, 13]. Recently, several studies extended MI methods for imputing missing values in multilevel data [14, 15] and longitudinal data [4, 16, 17]. These extensions are also available in several software programs such as R [9, 18–26], Mplus [27], STATA [28, 29], Blimp [30], REALCOM-IMPUTE [31], SAS [32], and Stat-JR [33]. In addition, in 2013 and 2016, Genolini et al. introduced several SI approaches to impute monotone/dropout and non-monotone/intermittent missing data in longitudinal studies [34, 35].

A few studies compared MI approaches for imputing missing values in longitudinal studies [16, 17, 36]. These studies used parametric approaches like LMM as an analysis model of interest. However, it is uncertain how well the different MI approaches perform when the statistical model of interest is a non-parametric longitudinal model. In addition, there is no comparison of SI and MI approaches in the literature, where the target analysis is a non-parametric longitudinal model. Hence, the present study is the first to consider non-parametric estimation methods for longitudinal data analysis following missing data imputation with SI and MI approaches.

In this study, the non-parametric longitudinal method of interest is the longitudinal regression tree algorithm proposed by Sela et al. This algorithm is named the random effects expectation–maximization (REEM) tree algorithm [37].

The primary purpose of this study is to evaluate the performance of MI and SI approaches for imputing missing values in longitudinal data. The longitudinal data for this study were obtained from the longitudinal Tehran cardiometabolic genetic study (TCGS) to assess the association between diastolic/systolic blood pressure (DBP/SBP) and predictor variables such as age, gender, and body mass index (BMI).

## Methods

### Tehran cardiometabolic genetic study (TCGS)

Subjects of the study are extracted from TCGS, an ongoing cohort study based on the framework of the Tehran Lipid and Glucose Study (TLGS). TLGS is the first prospective cohort study in West Asia, and was conducted in Tehran, the capital of Iran. This study was designed to assess the epidemiology of non-communicable diseases of participants from district 13 of Tehran with 24 years of follow-up. The first or baseline phase of the TLGS study was started in February 1999, and the individuals were selected via a multistage stratified cluster random sampling method with follow up every three years. The primary purpose of the TLGS is to determine the prevalence of cardiovascular diseases (CVD) and risk factors for the Tdevelopment of these diseases. The design of the TLGS study has been reported elsewhere [38–42].

**Fig. 1:**
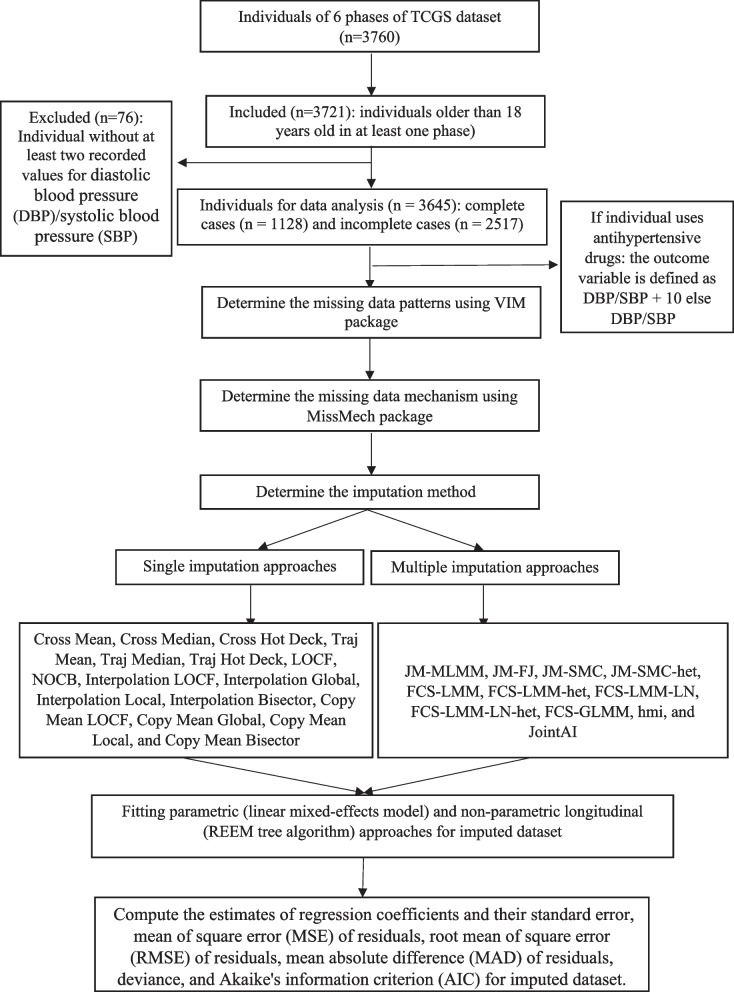
The study structure for the selection of individuals and statistical analysis

In the present study, some participants of TCGS were used to evaluate the association between DBP/SBP and predictor variables such as age, sex, and BMI [43, 44]. The predictor variables such as sex and age had no missing values (Table S1 in supplementary file provides sample data for the first 20 individuals of TCGS). However, the BMI and outcome variables (DBP/SBP) had missing values in all six waves of the TCGS study. SI and MI approaches were used to impute missing values of incomplete variables. Parametric linear mixed-effects and non-parametric longitudinal regression tree models were used for longitudinal data analysis after imputing missing data. The study structure for selecting individuals and statistical analysis plan is shown in Fig. 1. In the following sections, each step for data analysis is fully described.

### Missing data mechanisms

Three missing data mechanisms are defined for generating missing data: missing completely at random (MCAR), missing at random (MAR), and missing not at random (MNAR [3]. We can distinguish between the mechanisms by defining outcome Y = (Y^observed^, Y^missing^) and missing indicator R (1: observed and 0: missing). Each missing data mechanism suggests a general imputation approach.

If missingness probability is not related to observed and unobserved data, then data are MCAR. According to this mechanism, the distribution of missing data P $\left(\mathrm{R}|\mathrm{Y}\right)$ = P(R). While this assumption is unrealistic, the listwise deletion method is an unbiased method for dealing with missing data when this assumption is established. Multiple imputation (MI) approaches are developed under the MAR assumption, which states that the probability of missingness is related to the observed data and is not dependent on the unobserved data. In this case, the distribution of missing data is defined as P $\left(\mathrm{R}|\mathrm{Y}\right)$ = P $\left(\mathrm{R}|{\mathrm{Y}}^{\mathrm{observed}}\right)$, and MI methods can generate unbiased and efficient results [45]. Traditional models for longitudinal data analysis like GEE and models based on the maximum likelihood estimation like GLMMs also lead to valid estimates when missing mechanisms are MCAR and MAR, respectively [46].

The MNAR mechanism occurs when the probability of missingness is related to observed and unobserved data, or the distribution of missing data is equal to P $\left(\mathrm{R}|{\mathrm{Y}}^{\mathrm{observed}},{\mathrm{Y}}^{\mathrm{missing}}\right)$. Selection and pattern-mixture models have been introduced to handle MNAR data [47]. Assuming that the mechanism is MCAR is a very strong assumption; there are several tests to assess this assumption against not MCAR [48, 49]. The MAR assumption is most common in practice, and this can be tested against the MCAR assumption. However, it is never possible to rule out the assumption of MNAR. The missing data mechanism of multiple variables may be a mixture of any or all mechanisms described here.

### Missing data patterns

In longitudinal studies, missing values are based on non-monotone/intermittent and monotone/dropout processes [50]. The non-monotone pattern is created when study information is not available for a subject at one time point, but the subject returns at a subsequent time point. A monotone pattern is unlike a non-monotone pattern; if a subject misses a particular follow-up, then this subject is not available again. In practice, these two patterns can occur together for different measures.

### SI approaches to impute missing values in longitudinal data

In SI approaches, a single value is estimated for each missing data point. In 2013 and 2016, Genolini et al. introduced several SI approaches to impute monotone/dropout and non-monotone/intermittent missing data in longitudinal studies [34, 35].

To understand the computational strategy related to this section, a data set of *n* clusters (subjects) is considered. A time-dependent variable is recorded at *t* time points for each cluster. In this case, a trajectory for cluster *i* and a cross-sectional measurement for a particular time point *k*, is defined as the sequence y_i._ = (y_i1,_ y_i2,_ …, y_it_) and as the vector y_.k_ = (y_1k,_ y_2k,_ …, y_nk_), respectively. Let y_ik_ show a missing value for cluster *i* at a specific time point *k*. y_ik_ is non-monotone missing if time points as *a* < *k* < *b* exists and y_ia_ and y_ib_ are not missing. y_ik_ is monotone missing if for all time points *h* > *k*, y_ih_ is missing.

SI methods are classified into three imputation classes: cross-sectional (methods such as cross-mean, cross-median, and cross-hot deck), longitudinal (methods such as traj-mean, traj-median, traj-hot deck, last observation carried forward (LOCF), next observation carried backwards (NOCB), interpolation LOCF, interpolation global, interpolation local, and interpolation bisector), and cross-sectional-longitudinal (methods such as copy mean LOCF, copy mean global, copy mean local, and copy mean bisector). The cross-sectional imputation methods deal with observed data at a specific time (across clusters) for replacing the missing values in this time, whereas longitudinal imputation methods deal with observed data of the same cluster to impute the missing values in this cluster. The cross-sectional-longitudinal imputation methods utilize both cross-sectional information (y_i._) and longitudinal information (y_.j_).

### Cross methods

The cross methods include cross-mean, cross-median, and cross-hot deck. In the cross-mean method, y_ik_ at a particular time point is estimated by the mean of all values observed at the time point of interest. Likewise, the cross-median method uses the median of all values observed at the time point of interest instead. In the cross-hot deck method, y_ik_ at a particular time point is estimated by using a randomly selected value observed at the time point of interest.

### Traj methods

The traj methods include traj-mean, traj-median, and traj-hot deck. In the traj-mean method, y_ik_ is estimated by the mean of all values observed at the trajectory of interest y_i_. The traj-median method uses the median of all values observed at the trajectory of interest. In the traj-hot deck method, y_ik_ at a particular time point is estimated by a randomly selected value observed from the trajectory interest.

### LOCF (Last Occurrence Carried Forward) and NOCB (Next Occurrence Carried Backward)

In the LOCF and NOCB methods, y_ik_ is estimated by the last and next observed value of the trajectory of interest, respectively.

### Interpolation methods

Interpolation methods include the four methods: interpolation-LOCF, interpolation-global, interpolation-local, and interpolation-bisector. In all interpolation methods, a non-monotone missing (y_ik_) is replaced by drawing a line between the values immediately surrounding y_ik,_ and the mathematical formula of this step is as follows: ${\mathrm{y}}_{\mathrm{ia}}+(\mathrm{k}-\mathrm{a})\frac{{\mathrm{y}}_{\mathrm{ib}}-{\mathrm{y}}_{\mathrm{ia}}}{(\mathrm{b}-\mathrm{a})}$ (y_ia_ and y_ib_ are values immediately surrounding y_ik_). But these methods have different strategies to deal with monotone missing values. For example, the interpolation-LOCF uses LOCF or NOCB methods to solve this problem. In the interpolation-global method, replacing a monotone missing is performed by drawing a line linking the first and the last observed values. In the interpolation-local method, monotone missing values are generated first by drawing a line linking the first and second non-missing value. Then, monotone missing values at the end of the trajectory are replaced by drawing a line linking the last and penultimate non-missing value. Finally, the interpolation-bisector method provides an intermediate method using the bisector of interpolation-global and interpolation-local for imputing monotone missing values, and the imputed values are chosen on the bisectors.

### Copy-mean methods

The copy-mean methods include copy-mean LOCF, copy-mean global, copy-mean local, and copy-mean bisector. The copy-mean LOCF method is based on two steps for the imputation of missing observations. First, missing values are imputed using the LOCF method to provide an initial approximation of these values. Then the mean trajectory of the population is used to refine the initial approximation in the previous step. Let,$({\overline{\mathrm{y}} }_{.1}, \dots ,{\overline{\mathrm{y}} }_{.\mathrm{t}})$: the mean trajectory of a populationy_ik:_ the first missing value of i^th^ trajectory${\mathrm{y}}_{\mathrm{ik}}^{\mathrm{LOCF}}$: the imputed value for y_ik_ using LOCF method for all time points k ≥ d$\left({\overline{y} }_{.1}^{\mathrm{LOCF}}, \dots ,{\overline{\mathrm{y}} }_{.\mathrm{t}}^{\mathrm{LOCF}}\right)$: the mean trajectory of a population with missing values using the LOCF methodAV_k_: the average variation at k^th^ time point and is equal to ${\overline{y} }_{.k}-{\overline{\mathrm{y}} }_{.\mathrm{k}}^{\mathrm{LOCF}}$

The missing value y_ik_ is obtained from the copy mean LOCF by adding AV_k_ to the imputed value for y_ik_ using the LOCF method $\left({\mathrm{y}}_{\mathrm{ik}}^{\mathrm{LOCF}}+{\mathrm{AV}}_{\mathrm{k}}\right)$

The computational strategies of the copy-mean local, copy-mean global, and copy-mean bisector are similar to the copy-mean LOCF method, except these methods use the interpolation-local method, interpolation-global method, and interpolation-bisector method to provide an initial approximation of missing values, respectively.

### MI approaches to impute missing values in longitudinal data

MI approaches were proposed by Rubin in 1987 [51], and are flexible and popular methods for imputing missing values based on three steps: imputation step, analysis step, and pooling step [52, 53]. MI approaches use a Bayesian strategy, where posterior estimation is conducted using the Markov Chain Monte Carlo (MCMC) method. These approaches can be decomposed into two approaches, JM and FCS, described in subsequent sections.

In the imputation step, missing data are estimated several times by sampling through their posterior predictive distribution given the observed data and the parameter values of the imputation model. In the next step, multiple complete data sets produced via the imputation step are analyzed using the statistical model of interest. Finally, the results obtained from the analysis step, such as the estimates of regression coefficients with their standard error and criteria of predictive performance, are pooled using Rubin's rules to account for the uncertainty of the imputation [3, 51]. This process is shown graphically in Figure S1 (supplementary file).

Generating more than ten datasets positively affect statistical power [54]; Enders (2010) argues that generating 20 multiple imputed datasets is appropriate [55]. Based on this, we generated 20 multiple datasets of imputed data to impute missing data in the TCGS study using MI approaches.

MI approaches have an attractive property because auxiliary variables can be included in the imputation step without being in the analysis step. Auxiliary variables provide information about missing data and improve the quality of missing data imputation [45]. Researchers also can use a larger number of these variables to reduce the negative effects of MNAR [26].

The MI approaches are based on the MAR assumption, and the inclusion of auxiliary variables in the imputation step raises the plausibility of this assumption. The imputation model should include all the estimations in the analysis step in these imputation approaches. Otherwise, the analysis may generate biased estimates. In real applications, imputation models with many variables can lead to problems such as multi-collinearity and non-convergence [26].

In the present study, we used a number of MI approaches available in R software for missing data imputation; these approaches include joint multivariate LMM (JM-MLMM) [4], joint multivariate LMM with heteroskedastic covariance matrices across all clusters (JM-MLMM-het) [56], full joint (JM-FJ) [57], substantive model compatible joint modelling approach (JM-SMC) [58], substantive model compatible joint modelling approach with heteroskedastic covariance matrices across all clusters (JM-SMC-het) [59], FCS-LMM [26], FCS-LMM with heteroskedastic residual variance across all clusters (FCS-LMM-het) [26], FCS-LMM-latent normal (FCS-LMM-LN) [60], FCS-LMM-LN with heteroskedastic residual variance across all clusters (FCS-LMM-LN-het) [60], FCS-MLMM with latent normal variables (FCS-MLMM-LN) [22], hierarchical multiple imputation (hmi) [23], and joint analysis and imputation (JointAI) [61]. Each approach is explained in the following sections. In addition, we mention the number of iterations, burn-in period, and convergence criteria for each MI approach.

### JM-MLMM method

Schafer and Yucel (2002) introduced the JM-MLMM method for imputing missing values in longitudinal data using joint multivariate linear mixed models (JM-MLMM) instead of treating time-dependent variables as distinct variables for imputing variables with missing values. In this method, qualitative variables are imputed as continuous or dummy variables. The JM-MLMM method also assumes that random effects are based on a normal distribution with constant covariance matrices across all subjects (clusters) [4].

### JM-FJ method

In real applications, longitudinal data with missing values may be a mixture of qualitative and quantitative variables, so the normality assumption for these incomplete variables may be unrealistic. Goldstine et al. (2009) suggested the JM-MLMM-LN method based on the JM-MLMM method, which includes latent normal (LN) variables for imputing a mixture of normal and non-normal variables [57]. Asparouhov and Muthen (2010) proposed the JM-FJ method based on the JM-MLMM-LN method to impute missing values in longitudinal data using all variables in the imputation process as outcome variables.

These two methods and the JM-MLMM method are implemented in package mitml [19]. The potential scale reduction criterion near 1 or < 1.05 for all parameters and diagnostics plots were used to assess the convergence. If the potential scale reduction criterion was larger than 1.05, the iterations of the burn-in period were increased.

### JM-SMC and JM-SMC-het methods

Goldstine et al. (2014) extended the JM-MLMM-LN method to the JM-SMC method by defining the joint imputation method as the product of the analysis model and the joint distribution of variables [58]. The JM-SMC method can also accommodate random covariance matrices across all subjects, and this method is defined as the JM-SMC-het method. These methods use diagnostics plots to assess convergence.

In the present study, all JM approaches were conducted based on the 1000 iterations and 5000 iterations for a burn-in period to establish the stability of parameters distribution.

### FCS-LMM and FCS-LMM-het methods

FCS-LMM is an FCS adaptation of the JM-MLMM method, proposed by van Buuren et al. (2011). This method fits a multilevel LMM to impute missing values of incomplete variables conditional to other variables, cycling iteratively based on the univariate imputation models. In this method, qualitative variables are imputed as continuous variables or as dummy variables. The FCS-LMM method assumes normal distributions for all variables with missing values and a fixed residual variance across all subjects [26]. Van Buuren (2011) extended the FCS-LMM method to the FCS-LMM-het method to deal with heteroskedastic residual variance across all clusters [14].

### FCS-LMM-LN and FCS-LMM-LN-het methods

Enders et al. (2017) suggested the FCS-LMM-LN method by extending the FCS-LMM method to LN variables [60]. This method imputes missing data using a value randomly selected from observed values having the nearest predicted mean based on the LMM to particular missing data. In the FCS-LMM-LN-het method, the continuous variables with missing values are imputed using a LMM.

### FCS-MLMM-LN method

Audigier and Resche-Rigon modified the JM-MLMM-LN approach to impute missing observations based on an FCS framework where only one variable is considered missing at a time [22]. At each step, all of the variables in the imputation model are considered as outcomes (one of variables in the imputation model is treated as incomplete variable and the rest are considered as complete variables). Using this approach, the incomplete binary and categorical variables are imputed using latent normal variables as for JM-MLMM-LN.

All FCS approaches mentioned were conducted based on the 20 iterations and 5 iterations for a burn-in period to establish the stability of parameters distribution (In these approaches, the convergence of estimations can occur with 5 or 10 iterations). In addition, diagnostic graphs were used to examine convergence [26].

### Non-parametric longitudinal analysis method

The longitudinal tree-based methods are non-parametric methods for analyzing longitudinal data. Medical studies have used these methods to determine disease risk factors and identify high- and low-risk subgroups of patients [62, 63] by extracting homogeneous subgroups of observations that can be appropriately used for subgroup analysis [64]. Since most studies evaluating longitudinal changes in the outcome variable are conducted in the context of a heterogeneous population, traditional parametric longitudinal models might not provide a good fit and could potentially result in biased estimates. In addition, the actual values of the model parameters may differ between homogeneous subgroups. Because the tree-based models can extract homogeneous subgroups of observations and estimate heterogeneous treatment effects, they may be better positioned to assist the clinician in decision-making [65, 66].

Unlike traditional parametric longitudinal models, these methods do not require assumptions about the functional form of the data and are robust to outliers and multicollinearity. They can accommodate non-linear relationships and high-order interactions. The monotone transformations of predictor variables do not have any effect on the results. The interpretation of tree methods is straightforward because the results are shown graphically [67–70].

The classification and regression tree (CART) algorithm is the best-known tree algorithm for cross-sectional data modeling [71]. Sela et al. (2012) extended this tree algorithm for longitudinal data by combining the LMM and the CART algorithm. This longitudinal regression tree algorithm is named the random effects expectation–maximization (REEM) tree algorithm [37].

The previous section described how the LMM uses a parametric linear form for fixed-effects; this form cannot easily handle complex non-linear relationships or datasets with very large numbers of predictor variables. The REEM tree algorithm solves this problem using a non-parametric method like the CART algorithm to estimate the fixed effects. The estimation method of REEM is as follows:

1. Set the initial values equal to zero for ${\widehat{b}}_{i}$.

2 Run the following steps until the convergence of ${\widehat{b}}_{i}$ (convergence is established when change in the likelihood or restricted likelihood < predetermined tolerance value (e.g. 0.001)).a) Fit a regression tree to estimate an initial approximation of $f$ using the CART algorithm, based on response variable,${y}_{it}- {Z}_{it}{\widehat{b}}_{i}$, predictor variables,${x}_{it}=({x}_{it1}, \dots , {x}_{itK})$, for i = 1, …, I and t = t = 1, …,${T}_{i}$. This regression tree generates a set of predictor variables, I(${x}_{it}\in {\mathrm{g}}_{\mathrm{p}}$), where ${\mathrm{g}}_{\mathrm{p}}$ ranges over all terminal nodes of the tree.b) Run the LMM, ${y}_{it}={Z}_{it}{b}_{i}+\sum_{p}\mathrm{I}\left({x}_{it}\in {\mathrm{g}}_{\mathrm{p}}\right){\mu }_{p}+{\varepsilon }_{it}$, to estimate ${\widehat{b}}_{i}$ from the fitted model.

3 Use estimated predicted response ${\widehat{\mu }}_{p}$ from the fitted LMM in step 2b instead of the predicted response at each terminal node of tree.

### Simulation study

Performance of the various imputation approaches was compared using simulation data. We generated 1000 datasets, each of which included 1000 individuals, mimicking the TCGS data. In each simulated dataset, variables were generated as follows (all parameters in the data generating models were estimated from the original data to ensure that the simulated datasets were comparable to a real data example):

1. Sex variable was generated using a binomial distribution with probabilities 0.5.

2 Age variable at the first wave was generated using a truncated normal distribution with exact minimum = 1, maximum = 84, mean = 39.34, and standard deviation = 16.23. Age at other waves was generated as follows:

$$\begin{array}{cc}{\mathrm{Age}}_{\mathrm{ij}}= {\mathrm{Age}}_{\mathrm{i}1}+\left(\mathrm{j}-1\right)\times 3\mathrm{ i}\hspace{0.17em}=\hspace{0.17em}1, \dots , 3645,\mathrm{ j}\hspace{0.17em}=\hspace{0.17em}2, \dots , 6& \mathrm{i}\hspace{0.17em}=\hspace{0.17em}1, \dots , 3645,\mathrm{ j}\hspace{0.17em}=\hspace{0.17em}2, \dots , 6\end{array}$$

3 The main predictor variable (BMI) at each wave was generated based on age and sex as well as individual-level random effects and individual-level noise in each wave:

$$\begin{array}{cc}{\mathrm{BMI}}_{\mathrm{ij}}=19.86+0.136\times {\mathrm{Age}}_{\mathrm{ij}}+2.360 \times {\mathrm{Sex}}_{\mathrm{i}}+ {\mathrm{\varnothing }}_{0\mathrm{i}}+ {\mathrm{\varnothing }}_{\mathrm{ij}}& \mathrm{i}=\hspace{0.17em}1, \dots ., 3645,\mathrm{ j}\hspace{0.17em}=\hspace{0.17em}1, \dots , 6\end{array}$$

where ${\mathrm{\varnothing }}_{0\mathrm{i}}$ = N (0, 4.50) is the random intercept and ${\mathrm{\varnothing }}_{\mathrm{ij}}$ = N (0, 1.57) is the residual error.

4 The outcome variable, DBP at each wave was generated using the following linear process:

$$\begin{array}{l}{\mathrm{DBP}}_{\mathrm{ij}}=55.03+0.098\times {\mathrm{Age}}_{\mathrm{ij}}-3.434 \times {\mathrm{Sex}}_{\mathrm{i}}+ {0.707 {\times \mathrm{ BMI}}_{\mathrm{ij}}+\upgamma }_{0\mathrm{i}}+ {\upgamma }_{\mathrm{ij}}\\ \mathrm{i}\hspace{0.17em}=\hspace{0.17em}1, \dots ., 3645,\mathrm{ j}\hspace{0.17em}=\hspace{0.17em}1, \dots , 6\end{array}$$

where $\gamma$_0i_ = N (0, 6.35) is the random intercept and ${\upgamma }_{\mathrm{ij}}$= N (0, 6.88) is the residual error.

After generating simulated data, missingness for some observations of BMI and DBP are generated based on the MAR mechanism. The following equations are used:

$$\mathrm{logit }\left(\mathrm{P}\left({\mathrm{BMI}}_{\mathrm{ij}}=\mathrm{missing}\right)\right)={\beta }_{0\mathrm{j}}+{\beta }_{1\mathrm{j}}{\mathrm{Age}}_{\mathrm{ij}}+{\beta }_{2\mathrm{j}}{\mathrm{DBP}}_{\mathrm{ij}}$$

$$\mathrm{logit }\left(\mathrm{P}\left({\mathrm{DBP}}_{\mathrm{ij}}=\mathrm{missing}\right)\right)={\varphi }_{0\mathrm{j}}+{\varphi }_{1\mathrm{j}}{\mathrm{Age}}_{\mathrm{ij}}+{\varphi }_{2\mathrm{j}}{\mathrm{BMI}}_{\mathrm{ij}}$$

The parameters $\beta$_0j,_ $\beta$_1j,_ $\beta$_2j,_ $\varphi$_0j,_ $\varphi$_1j,_ and $\varphi$_2j_ were determined based on the TCGS data to ensure a similar proportion of missing data for each variable at each wave; the proportions in both TCGS study and simulation study are shown in Table 1. These parameters are as follows:

**Table 1:** The proportions of missing data in both TCGS study and simulation study

Phase of the TCGS study	Missing data proportions in BMI		Missing data proportions in DBP	
	TCGS study	Simulation study	TCGS study	Simulation study
1	0.252	0.252	0.249	0.248
2	0.266	0.266	0.230	0.230
3	0.189	0.190	0.184	0.184
4	0.168	0.168	0.144	0.144
5	0.232	0.232	0.195	0.195
6	0.329	0.327	0.297	0.298

$${\upbeta }_{0}=\left\{-2.646, -2.634, -3.047, -3.271, -2.872, -2.440\right\}$$

$${\varphi}_{0}=\left\{-1.701, -1.815, -2.091, -2.386, -2.104, -1.522\right\}$$

$${\upbeta }_{1}={\varphi}_{1}=0.002$$

$${\upbeta }_{2}={\varphi}_{2}=0.02$$

### Criteria for comparing the performance of missing data imputation methods

The performance of imputation approaches under the MAR mechanism and statistical methods applied to the real and simulated data sets was compared by evaluating the standard errors of regression coefficients (SE), MSE, RMSE, MAD, deviance, and AIC. The imputation approaches with smaller value in terms of SE, MSE, RMSE, MAD, deviance, and AIC indicate better performance.

### Software programs

R software was used to impute missing longitudinal data and data analysis, and R packages used are mentioned in Table 2. The R codes of SI and MI approaches for missing data imputation and data simulation are available in https://github.com/MinaJahangiri/R-codes-of-missing-imputation-methods.

**Table 2:** R packages for data analysis

Step	Method	R package	Reference
Missing data pattern	Graphically	VIM	[80]
MI approaches for missing data imputation	JM-FJ	mitml	[19]
	JM-MLMM		
	JM-SMC	jomo	[18]
	JM-SMC-het		
	hmi	hmi	[23]
	JointAI	JointAI	[24]
	FCS-LMM	mice	[8]
	FCS-LMM-het		
	FCS-LMM-LN	miceadds	[20]
	FCS-LMM-LN-het		
	FCS-MLMM-LN	micemd	[22]
SI approaches for missing data imputation	Cross Mean	longitudinalData	[25]
	Cross Median		
	Cross Hot Deck		
	Traj Mean		
	Traj Median		
	Traj Hot Deck		
	LOCF		
	NOCB		
	Interpolation LOCF		
	Interpolation Global		
	Interpolation Local		
	Interpolation Bisector		
	Copy mean LOCF		
	Copy mean global		
	Copy mean local		
	Copy mean bisector		
Fitting parametric longitudinal model	Linear mixed effects model	lme4	[81]
Fitting non-parametric longitudinal model	Longitudinal regression tree	REEMtree	[82]
Missing data simulation	genMiss function	simstudy	[83]

## Results

**Fig. 2:**
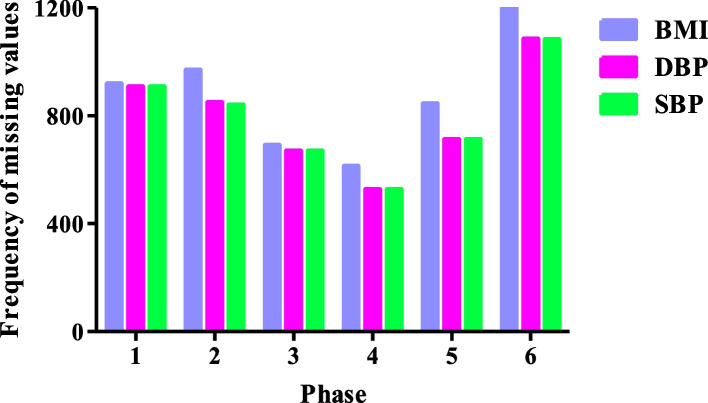
Frequency of missing values for variables like body mass index (BMI), diastolic blood pressure (DBP), and systolic blood pressure (SBP) at each phase of the TCGS study (the frequency of missing values for BMI variable at each phase of the TCGS study are 918, 969, 690, 612, 844, and 1199, respectively, the frequency of missing values for DBP variable at each phase of the TCGS study are 907, 849, 669, 526, 712, and 1084, respectively, and the frequency of missing values for SBP variable at each phase of the TCGS study are 907, 840, 669, 526, 712, and 1082, respectively)

The study's variables with missing values are BMI (predictor variable) and DBP/SBP (outcome variables). Figure 2 shows the frequency of missing values for these variables at each phase of the TCGS study. The descriptive statistics of TCGS data are shown in the Table S2 (supplementary file). The percentage of missing values in a particular combination of variables based on the long data format is visually shown in Figure S2 (supplementary file). Seventy-six percent observations have no missing values, and 21% have missing values for BMI, DBP, and SBP, simultaneously. The missing data pattern of TCGS data indicated that both monotone and non-monotone patterns were present. The formal MCAR test indicated that the MCAR assumption is not reasonable (P < 0.001, so the MCAR assumption is rejected at a significance level of 0.05).

After the imputation of missing values, the data modeling results based on the LMM and REEM longitudinal tree algorithm for the two outcome variables (DBP and SBP) are shown in Tables 3, 4, 5 and 6. According to Tables 3 and 5, all SI approaches except cross-median, and cross-hot deck performed similarly with respect to estimates of regression coefficients and their standard error estimates.

**Table 3:** Results of linear mixed effects model for diastolic blood pressure (DBP)

Imputation method	$\beta$(SE)					MSE	RMSE	MAD	Deviance	AIC
	Intercept	Age	Sex	BMI	Time					
Complete cases	55.563 (1.124)	0.075 (0.016)	-2.778 (0.462)	0.706 (0.038)	-0.024 (0.074)	0.861	0.928	0.716	48345.57	48343.45
Interpolation LOCF	54.231 (0.617)	0.124 (0.008)	-3.238 (0.278)	0.692 (0.021)	-0.063 (0.039)	0.854	0.924	0.699	155033.2	155025.3
Interpolation global	54.38 (0.646)	0.123 (0.009)	-3.387 (0.298)	0.689 (0.022)	-0.118 (0.046)	0.859	0.927	0.671	162086.5	162079.5
Interpolation local	54.050 (0.676)	0.122 (0.009)	-3.373 (0.309)	0.703 (0.023)	-0.069 (0.049)	0.864	0.930	0.652	167074.2	167067.7
Interpolation bisector	54.285 (0.649)	0.123 (0.009)	-3.365 (0.299)	0.692 (0.022)	-0.108 (0.045)	0.860	0.927	0.668	162527.8	162520.9
copyMean.LOCF	54.670 (0.613)	0.126 (0.008)	-3.237 (0.277)	0.684 (0.022)	-0.119 (0.039)	0.854	0.924	0.702	155136.1	155128.3
copyMean.global	54.672 (0.638)	0.125 (0.009)	-3.395 (0.294)	0.690 (0.022)	-0.161 (0.044)	0.859	0.927	0.677	161560.7	161553.6
copyMean.local	54.851 (0.666)	0.120 (0.009)	-3.356 (0.305)	0.696 (0.023)	-0.183 (0.048)	0.865	0.930	0.657	166566.1	166559.5
copyMean.bisector	54.679 (0.641)	0.124 (0.009)	-3.369 (0.295)	0.692 (0.022)	-0.165 (0.044)	0.860	0.927	0.674	162051.4	162044.3
LOCF	54.507 (0.609)	0.125 (0.009)	-3.145 (0.278)	0.679 (0.021)	-0.073 (0.039)	0.854	0.924	0.702	155864.4	155856.5
NOCB	54.002 (0.627)	0.124 (0.009)	-3.326 (0.281)	0.702 (0.022)	-0.058 (0.040)	0.855	0.925	0.706	155878.9	155871.2
Traj mean	54.407 (0.615)	0.120 (0.008)	-3.20 (0.275)	0.707 (0.021)	-0.159 (0.037)	0.854	0.924	0.701	152373.1	152365
Traj median	54.675 (0.614)	0.120 (0.008)	-3.223 (0.274)	0.697 (0.021)	-0.160 (0.037)	0.852	0.923	0.653	152671	152662.9
Traj hot deck	56.433 (0.618)	0.125 (0.009)	-3.064 (0.279)	0.617 (0.021)	-0.136 (0.040)	0.852	0.923	0.659	156228.2	156220.4
Cross mean	54.847 (0.521)	0.090 (0.007)	-2.357 (0.214)	0.717 (0.018)	-0.091 (0.037)	0.855	0.925	0.707	155407.2	155397.6
Cross median	54.683 (0.522)	0.092 (0.007)	-2.347 (0.215)	0.711 (0.018)	-0.006 (0.037)	0.872	0.934	0.705	155540	155530.4
Cross hot deck	60.838 (0.512)	0.106 (0.007)	-1.902 (0.230)	0.452 (0.016)	-0.010 (0.043)	0.873	0.934	0.708	162970.7	162961.8
FCS-LMM	53.535 (0.486)	0.118 (0.007)	-3.163 (0.224)	0.733 (0.016)	-0.111 (0.041)	0.883	0.940	0.717	161290.4	161281.1
FCS-LMM-het	53.535 (0.486)	0.118 (0.007)	-3.163 (0.224)	0.733 (0.016)	-0.111 (0.041)	0.882	0.939	0.721	161290.4	161281.1
FCS-MLMM-LN	53.472 (0.590)	0.118 (0.008)	-3.216 (0.262)	0.734 (0.021)	-0.077 (0.040)	0.882	0.939	0.721	157445.7	157437.6
FCS-LMM-LN	53.723 (0.589)	0.121 (0.008)	-3.168 (0.265)	0.718 (0.021)	-0.065 (0.040)	0.861	0.928	0.721	157612.8	157604.8
FCS-LMM-LN-het	56.023 (0.510)	0.133 (0.008)	-2.874 (0.269)	0.601 (0.015)	-0.048 (0.041)	0.860	0.928	0.716	158355.2	158346.5
JointAI	56.660 (0.688)	0.119 (0.008)	-3.219 (0.264)	0.735 (0.021)	-0.087 (0.040)	0.860	0.928	0.720	157473.5	157465.4
hmi	53.409 (0.594)	0.118 (0.008)	-3.230 (0.264)	0.736 (0.021)	-0.077 (0.040)	0.860	0.927	0.720	157440	157432
JM-SMC	53.568 (0.594)	0.118 (0.008)	-3.204 (0.265)	0.730 (0.021)	-0.076 (0.040)	0.860	0.927	0.720	157451.2	157443.2
JM-SMC-het	53.562 (0.592)	0.119 (0.008)	-3.211 (0.264)	0.729 (0.021)	-0.077 (0.040)	0.860	0.928	0.720	157463.1	157455.1
JM-MLMM	53.739 (0.593)	0.120 (0.008)	-3.203 (0.264)	0.722 (0.021)	-0.094 (0.040)	0.860	0.928	0.720	157443.6	157435.5
JM-FJ	53.713 (0.593)	0.119 (0.008)	-3.229 (0.264)	0.726 (0.021)	-0.099 (0.040)	0.860	0.927	0.720	157381.1	157373.1

**Table 4:** Results of random effects expectation–maximization (REEM) tree algorithm for diastolic blood pressure (DBP)

Imputation method	MSE	RMSE	MAD	Deviance
Complete cases	0.860	0.927	0.715	48402.31
Interpolation LOCF	0.853	0.924	0.698	155130.7
Interpolation global	0.858	0.926	0.672	162242.6
Interpolation local	0.864	0.929	0.654	167328.3
Interpolation bisector	0.859	0.927	0.669	162677.8
copyMean.LOCF	0.854	0.924	0.701	155243.2
copyMean.global	0.858	0.927	0.678	161725.4
copyMean.local	0.863	0.929	0.658	166834.6
copyMean.bisector	0.859	0.927	0.675	162305.9
LOCF	0.854	0.924	0.705	155906.2
NOCB	0.854	0.924	0.701	155904.5
Traj mean	0.851	0.922	0.658	152664.4
Traj median	0.851	0.923	0.662	152885.9
Traj hot deck	0.854	0.924	0.706	156429.8
Cross mean	0.871	0.933	0.70	155292
Cross median	0.871	0.933	0.70	155181.4
Cross hot deck	0.882	0.939	0.718	162979.3
FCS-LMM	0.881	0.939	0.721	161444.4
FCS-LMM-het	0.881	0.939	0.721	161444.4
FCS-GLMM	0.860	0.927	0.720	157721.4
FCS-LMM-LN	0.859	0.927	0.716	157916.3
FCS-LMM-LN-het	0.860	0.927	0.720	158532
JointAI	0.859	0.927	0.720	157743
hmi	0.859	0.927	0.720	157716
JM-SMC	0.859	0.927	0.720	157732
JM-SMC-het	0.859	0.927	0.720	157756.6
JM-MLMM	0.859	0.927	0.720	157715
JM-FJ	0.859	0.927	0.720	157638.5

**Table 5:** Results of linear mixed effects model for systolic blood pressure (SBP)

Imputation method	$\beta$(SE)					MSE	RMSE	MAD	Deviance	AIC
	Intercept	Age	Sex	BMI	Time					
Complete cases	73.253 (1.813)	0.525 (0.027)	-4.428 (0.769)	0.920 (0.061)	-0.958 (0.117)	0.855	0.925	0.692	54066.32	54068.99
Interpolation LOCF	74.850 (0.980)	0.581 (0.014)	-3.982 (0.451)	0.843 (0.034)	-1.272 (0.061)	0.851	0.923	0.666	173646.4	173643.1
Interpolation global	74.144 (1.018)	0.581 (0.015)	-4.147 (0.479)	0.870 (0.035)	-1.303 (0.069)	0.856	0.925	0.637	180542.2	180539.8
Interpolation local	73.784 (1.057)	0.580 (0.015)	-4.075 (0.494)	0.893 (0.036)	-1.335 (0.075)	0.861	0.928	0.620	185290.3	185288.4
Interpolation bisector	74.20 (1.021)	0.581 (0.015)	-4.111 (0.480)	0.870 (0.035)	-1.315 (0.070)	0.856	0.925	0.634	180942.4	180940
copyMean.LOCF	75.411 (0.974)	0.582 (0.014)	-3.964 (0.451)	0.827 (0.034)	-1.299 (0.061)	0.851	0.923	0.667	173696.2	173693
copyMean.global	74.655 (1.019)	0.583 (0.015)	-4.139 (0.479)	0.858 (0.035)	-1.318 (0.069)	0.856	0.925	0.639	180,559.1	180,556.6
copyMean.local	74.548 (1.057)	0.578 (0.015)	-4.057 (0.493)	0.881 (0.036)	-1.406 (0.075)	0.861	0.928	0.621	185334.4	185332.5
copyMean.bisector	74.748 (1.022)	0.582 (0.015)	-4.104 (0.480)	0.859 (0.035)	-1.342 (0.070)	0.857	0.925	0.636	180985.1	180982.7
LOCF	75.168 (0.963)	0.580 (0.014)	-3.888 (0.450)	0.829 (0.033)	-1.274 (0.062)	0.851	0.923	0.667	174341.2	174338
NOCB	74.192 (0.993)	0.581 (0.014)	-4.103 (0.456)	0.872 (0.034)	-1.281 (0.062)	0.852	0.923	0.672	174379	174375.9
Traj mean	74.688 (0.977)	0.575 (0.014)	-4.105 (0.448)	0.876 (0.033)	-1.376 (0.059)	0.852	0.923	0.670	170,867.3	170863.8
Traj median	74.849 (0.976)	0.577 (0.014)	-4.141 (0.447)	0.867 (0.033)	-1.380 (0.059)	0.849	0.921	0.629	171163.8	171160.3
Traj hot deck	77.092 (0.978)	0.583 (0.014)	-3.828 (0.454)	0.767 (0.033)	-1.360 (0.062)	0.849	0.922	0.633	174543.7	174540.5
Cross mean	77.646 (0.858)	0.432 (0.011)	-3.145 (0.361)	0.974 (0.029)	-1.203 (0.060)	0.852	0.923	0.677	176339.4	176334.8
Cross median	76.462 (0.858)	0.438 (0.011)	-3.022 (0.361)	0.971 (0.029)	-1.116 (0.060)	0.869	0.932	0.692	176435.9	176431.3
Cross hot deck	86.703 (0.855)	0.453 (0.012)	-2.492 (0.386)	0.599 (0.027)	-1.220 (0.071)	0.869	0.932	0.684	185,241.9	185,238.1
FCS-LMM	74.898 (0.783)	0.586 (0.011)	-3.875 (0.372)	0.799 (0.026)	-1.075 (0.065)	0.882	0.939	0.690	180754.4	180750
FCS-LMM-het	74.898 (0.783)	0.586 (0.011)	-3.875 (0.372)	0.799 (0.026)	-1.075 (0.065)	0.875	0.936	0.692	180754.4	180750
FCS-GLMM	73.635 (0.950)	0.577 (0.013)	-4.049 (0.434)	0.864 (0.033)	-1.014 (0.062)	0.875	0.936	0.692	176091.8	176088.5
FCS-LMM-LN	74.565 (0.924)	0.579 (0.013)	-3.880 (0.439)	0.825 (0.031)	-1.010 (0.063)	0.856	0.925	0.70	176,335.7	176,332.3
FCS-LMM-LN-het	84.718 (0.767)	0.615 (0.014)	-2.947 (0.448)	0.360 (0.018)	-0.899 (0.064)	0.856	0.925	0.691	177101.9	177097.4
JointAI	77.798 (1.120)	0.579 (0.013)	-4.035 (0.437)	0.855 (0.033)	-1.005 (0.063)	0.856	0.925	0.70	176190.7	176187.4
hmi	73.052 (0.954)	0.575 (0.013)	-4.140 (0.438)	0.892 (0.033)	-1.014 (0.063)	0.855	0.925	0.699	176055.4	176052.1
JM-SMC	73.771 (0.953)	0.578 (0.013)	-4.053 (0.437)	0.858 (0.033)	-1.023 (0.063)	0.856	0.925	0.699	176103.3	176100
JM-SMC-het	73.656 (0.953)	0.578 (0.013)	-4.085 (0.437)	0.861 (0.033)	-1.009 (0.063)	0.856	0.925	0.70	176120	176116.8
JM-MLMM	73.778 (0.949)	0.582 (0.013)	-4.135 (0.435)	0.880 (0.033)	-1.250 (0.062)	0.856	0.925	0.699	175978.9	175975.6
JM-FJ	73.810 (0.950)	0.581 (0.013)	-4.159 (0.436)	0.881 (0.033)	-1.254 (0.062)	0.856	0.925	0.699	175991.3	175988

**Table 6:** Results of random effects expectation–maximization (REEM) tree algorithm for systolic blood pressure (SBP)

Imputation method	MSE	RMSE	MAD	Deviance
Complete cases	0.854	0.924	0.689	54241.55
Interpolation LOCF	0.849	0.922	0.666	174247.8
Interpolation global	0.854	0.924	0.639	181020.8
Interpolation local	0.859	0.927	0.624	185848
Interpolation bisector	0.854	0.924	0.636	181482.6
copyMean.LOCF	0.849	0.922	0.667	174344.9
copyMean.global	0.854	0.924	0.641	181116.8
copyMean.local	0.859	0.927	0.624	186016.9
copyMean.bisector	0.855	0.924	0.638	181544.8
LOCF	0.850	0.922	0.672	174940.5
NOCB	0.850	0.922	0.671	175030.9
Traj mean	0.847	0.920	0.636	171740.2
Traj median	0.847	0.920	0.639	172071.6
Traj hot deck	0.850	0.922	0.677	175267.3
Cross mean	0.866	0.930	0.680	175619.2
Cross median	0.866	0.931	0.678	175871.9
Cross hot deck	0.881	0.939	0.692	185584.4
FCS-LMM	0.874	0.935	0.693	181290.1
FCS-LMM-het	0.874	0.935	0.693	181290.1
FCS-GLMM	0.854	0.924	0.70	176799.5
FCS-LMM-LN	0.854	0.924	0.692	176975
FCS-LMM-LN-het	0.854	0.924	0.701	177478.9
JointAI	0.854	0.924	0.70	176904.3
hmi	0.854	0.924	0.70	176767
JM-SMC	0.854	0.924	0.70	176812
JM-SMC-het	0.854	0.924	0.70	176818.3
JM-MLMM	0.854	0.924	0.699	176732.2
JM-FJ	0.854	0.924	0.699	176758.1

The results shown in Tables 3, 4, 5 and 6 indicated that parametric longitudinal models are not appropriate for analyzing the TCGS data. There appears to be non-linear relationships (for BMI variable) and the assumptions of homoscedasticity and normality of residuals are clearly not established by using the diagnostic plots such as standardized residuals versus BMI variable, quantile–quantile (QQ) plot of residuals, and plot of standardized residuals versus fitted values for linear mixed effects model, respectively. These assumptions for the LMM appear to have been violated regardless of the SI approach used; as an illustration, we show the diagnostic plots for the traj-mean method in Figures S3, S4, S5, S6, S7 and S8 (supplementary file). We compared the parametric and non-parametric models using SI approaches, and the parametric longitudinal models resulted in larger MSE, RMSE, and MAD than the longitudinal regression tree algorithm (Tables 3, 4, 5 and 6). Given these advantages of the non-parametric model, the comparison of SI and MI imputation approaches is only explained based on the REEM tree algorithm.

When comparing SI approaches that were used in conjunction with the REEM tree algorithm, traj-mean method performed the best (lowest MSE, RMSE, MAD, and deviance), and the cross-hot deck performed the worst (Tables 4 and 6). The tree structure of the REEM tree algorithm using traj-mean for imputation of missing values for two outcome variables, DBP and SBP, are shown in Figure S9 and Figure S10 (supplementary file).

Density plots of the observed and imputed data for incomplete variables like BMI, DBP, and SBP using mice packages are shown in Figure S11, S12, S13 and S14 (supplementary file). Figure S15 (supplementary file) also demonstrates the trace line plots of the mean and standard deviation of the imputed values against the iteration number for each replication. These trace lines are intermingled without any particular trend, so it appears estimation has converged. Due to space limitations, trace line plots for other FCS approaches are not shown, though these plots also indicated convergence. In addition, the Rhat statistic of mean and variances for all incomplete variables based on the FCS-LMM-LN and FCS-LMM-LN-het methods are near one, so the convergence of these methods is also established (Table S3 in supplementary file).

When comparing FCS approaches, the FCS-LMM-LN performed the best and FCS-LMM/FCS-LMM-het performed the worst (Tables 4 and 6). All of the JM approaches had a similar performance (Tables 4 and 6). The diagnostic plots of the JM approaches indicate convergence. In addition, the potential scale reduction criterion was near 1 for all parameters based on JM-FJ and JM-MLMM methods. Due to space limitations, these diagnostic plots are only included for the JointAI and JM-MLMM methods in Figures S16, S17, S18, S19 and S20 (supplementary file), respectively.

The simulation results were consistent with the real data analyses. The longitudinal regression tree algorithm provided better performance than the LMM for analyzing the simulated data under the missing at random (MAR) mechanism. In addition, the SI traj-mean method provided better performance (lowest MSE, RMSE, and MAD) than other imputation approaches (Tables 7 and 8). We have not assessed the bias, because the longitudinal tree algorithm, unlike LMM, does not generate the estimates of coefficient regression. Rather, we have based our evaluation of the methods on prediction performance.

**Table 7:** Simulation results of linear mixed effects model for diastolic blood pressure (DBP)

Imputation method	$\beta$(SE)					MSE	RMSE	MAD	Deviance	AIC
	Intercept	Age	Sex	BMI	Time					
Interpolation LOCF	57.12692 (1.036437)	0.110721 (0.01569074)	-3.200021 (0.4679745)	0.6076858 (0.03919466)	-0.03359099 (0.06541523)	0.8544829	0.9243823	0.7248456	41276.48	41274.17
Interpolation global	57.8273 (1.043371)	0.1157957 (0.01596507)	-3.105459 (0.4768267)	0.5686484 (0.0392315)	-0.0001583268 (0.06915446)	0.8574769	0.9260002	0.7163417	42164.38	42162.36
Interpolation local	58.88038 (1.066751)	0.1218176 (0.01650259)	-2.977433 (0.4939002)	0.5177882 (0.03952812)	-0.003048394 (0.0758609)	0.8625397	0.9287298	0.6967078	43542.53	43541.01
Interpolation bisector	58.06074 (1.037812)	0.1174172 (0.01595732)	-3.077177 (0.4769871)	0.556664 (0.03881613)	0.001809912 (0.06918759)	0.8574504	0.9259859	0.7163166	42168.54	42166.5
copyMean.LOCF	57.00081 (1.03387)	0.1103882 (0.01568596)	-3.204943 (0.4677538)	0.6099685 (0.03927595)	-0.001061418 (0.06535353)	0.8544874	0.9243847	0.7247638	41270.68	41268.38
copyMean.global	57.84495 (1.043165)	0.1158745 (0.0159612)	-3.102947 (0.4767275)	0.5678433 (0.03922722)	-0.001208831 (0.06915227)	0.8574909	0.9260078	0.7163547	42,164.87	42,162.85
copyMean.local	58.89496 (1.066593)	0.1218706 (0.01649862)	-2.97518 (0.4938004)	0.5172117 (0.03951998)	-0.004757409 (0.07585611)	0.862551	0.9287359	0.6967423	43542.18	43540.65
copyMean.bisector	58.06104 (1.042594)	0.1172312 (0.01600515)	-3.076642 (0.478326)	0.5575218 (0.03904693)	-0.005214816 (0.0696931)	0.857842	0.9261974	0.7140068	42280.49	42278.5
LOCF	57.49097 (1.038545)	0.1133142 (0.01577389)	-3.15695 (0.4707326)	0.5878046 (0.03922799)	-0.0334933 (0.06665705)	0.8555065	0.9249358	0.731262	41582.84	41580.63
NOCB	57.25775 (1.040266)	0.1114634 (0.0157608)	-3.184888 (0.470165)	0.6029563 (0.03917165)	-0.03643286 (0.06659423)	0.855514	0.9249398	0.7311435	41571	41568.78
Traj mean	57.10225 (1.029841)	0.109581 (0.0155384)	-3.218803 (0.4635713)	0.6164219 (0.03868269)	-0.0941321 (0.0631218)	0.8522359	0.9231661	0.6860667	40,610.16	40,607.64
Traj median	57.19844 (1.03154)	0.1102181 (0.01557755)	-3.210136 (0.4648093)	0.6117111 (0.03870845)	-0.09457664 (0.0634923)	0.8524687	0.9232922	0.6891027	40703.65	40701.17
Traj hot deck	58.0675 (1.037635)	0.1161083 (0.01574523)	-3.103703 (0.4702568)	0.5685524 (0.03868641)	-0.1016675 (0.06708686)	0.8558607	0.9251272	0.7329294	41655.91	41653.68
Cross mean	63.21342 (0.8601677)	0.1152429 (0.01234626)	-1.986146 (0.3750456)	0.3417965 (0.03072266)	0.09386702 (0.06364875)	0.8720438	0.9338323	0.7347808	41749.54	41745.65
Cross median	63.21735 (0.8601629)	0.1152627 (0.01234694)	-1.985775 (0.3750706)	0.3416021 (0.03071914)	0.09380551 (0.06365677)	0.8720513	0.9338364	0.7348175	41,751.37	41,747.48
Cross hot deck	65.66129 (0.8468008)	0.1266756 (0.01312589)	-1.786334 (0.4012122)	0.227812 (0.02789353)	0.1043702 (0.07495642)	0.8837707	0.9400898	0.7394809	43910.93	43907.67
FCS-LMM	60.28196 (0.5694889)	0.1331336 (0.01184806)	-2.799347 (0.3665625)	0.4434299 (0.009853248)	-0.001280831 (0.03963784)	0.983771	0.9918523	0.7720487	416,038.4	416,030
FCS-LMM-het	60.28196 (0.5694889)	0.1331336 (0.01184806)	-2.799347 (0.3665625)	0.4434299 (0.009853248)	-0.001280831 (0.03963784)	0.983771	0.9918523	0.7720487	416038.4	416030
FCS-MLMM-LN	55.42398 (0.7026658)	0.09829657 (0.01393327)	-3.508696 (0.4267242)	0.6937822 (0.01665573)	-0.004269101 (0.04404613)	0.9835628	0.9917473	0.7891817	399097.4	399090.7
FCS-LMM-LN	56.44262 (0.702904)	0.1067229 (0.01398748)	-3.264762 (0.4311401)	0.637727 (0.01595738)	-0.0006040621 (0.04430806)	0.9835589	0.9917454	0.7878218	399616.3	399609.5
FCS-LMM-LN-het	59.93189 (0.6904816)	0.1303412 (0.01413558)	-2.855235 (0.4366778)	0.4627641 (0.0135311)	-0.001121397 (0.04495049)	0.9835606	0.9917462	0.7861603	401764.5	401757.6
JointAI	55.81188 (0.7089093)	0.1023915 (0.01398527)	-3.343369 (0.4306244)	0.6695752 (0.01675999)	-0.0005635936 (0.04423095)	0.9835587	0.9917453	0.7880473	399310.9	399304.3
hmi	55.11064 (0.7092117)	0.09754292 (0.01400461)	-3.429275 (0.4312582)	0.705085 (0.01670312)	-0.001064226 (0.04428214)	0.9835572	0.9917445	0.7880315	399153.4	399146.8
JM-SMC	55.83689 (0.7088457)	0.1025074 (0.01398475)	-3.341177 (0.4306127)	0.668478 (0.01675367)	-0.001230324 (0.04423297)	0.9835589	0.9917454	0.7880463	399367.5	399360.8
JM-SMC-het	62.32773 (0.6864609)	0.1469084 (0.01442801)	-2.568779 (0.4466799)	0.3412525 (0.009964401)	-0.0003243688 (0.04582062)	0.9835423	0.991737	0.78781	400220.2	400212.4
JM-MLMM	55.80612 (0.709103)	0.1023837 (0.01399508)	-3.342461 (0.4309438)	0.669799 (0.01673468)	-0.001470212 (0.04426241)	0.9835584	0.9917451	0.7879724	399347.5	399340.8
JM-FJ	55.85205 (0.7090387)	0.1026766 (0.01398949)	-3.336058 (0.4307582)	0.6676159 (0.01675379)	-0.001963714 (0.04424547)	0.9835587	0.9917453	0.7880535	399345.1	399338.5

**Table 8:** Simulation results of random effects expectation–maximization (REEM) tree algorithm for diastolic blood pressure (DBP)

Imputation method	MSE	RMSE	MAD	Deviance
Interpolation LOCF	0.8527485	0.9234451	0.7243289	41396.3
Interpolation global	0.8555876	0.9249804	0.716464	42286.06
Interpolation local	0.8602081	0.9274721	0.6975486	43665.6
Interpolation bisector	0.8555478	0.9249548	0.7164404	42292.76
copyMean.LOCF	0.8528052	0.9234774	0.7245612	41397.91
copyMean.global	0.855585	0.9249841	0.7165564	42285.6
copyMean.local	0.8601975	0.9274707	0.6977025	43666.37
copyMean.bisector	0.8559014	0.925148	0.7141584	42402.8
LOCF	0.8537565	0.9239873	0.7304617	41693.62
NOCB	0.8537621	0.9239997	0.7302594	41683.84
Traj mean	0.8504812	0.9222132	0.6835874	40742.72
Traj median	0.8507346	0.9223542	0.687355	40833.42
Traj hot deck	0.8538656	0.9240471	0.7318596	41769.81
Cross mean	0.8693141	0.9323711	0.73382	41858.75
Cross median	0.8693081	0.9323664	0.7338405	41860.46
Cross hot deck	0.8799369	0.9380265	0.7386661	44022.77
FCS-LMM	0.9835786	0.9917558	0.7716194	416116.5
FCS-LMM-het	0.9835786	0.9917558	0.7716194	416116.5
FCS-GLMM	0.9833765	0.9916557	0.7890735	399160
FCS-LMM-LN	0.9833792	0.9916543	0.7874033	399770.8
FCS-LMM-LN-het	0.9833914	0.9916603	0.7856904	401880.2
JointAI	0.9833758	0.9916535	0.7875987	399447.3
hmi	0.9833762	0.9916535	0.7876294	399314.6
JM-SMC	0.9833776	0.991653	0.7876414	399511.8
JM-SMC-het	0.9833189	0.9916236	0.7871983	399572.9
JM-MLMM	0.9833767	0.9916536	0.7875398	399486.1
JM-FJ	0.9833752	0.9916533	0.787628	399484.6

## Discussion

Missing values are a significant problem in longitudinal studies, and managing this problem is essential. In the current study, we compared the performance of SI and MI approaches to impute longitudinal missing data in the context of using LMM and the REEM tree algorithm for data modelling. Previous studies have compared the performance of MI approaches when the statistical model of interest is a parametric longitudinal model; the performance of MI approaches when the statistical model of interest is a non-parametric longitudinal model is less well understood.

The current study provides a comprehensive assessment using missing imputation approaches for handling missing data in the TCGS dataset and simulated data under the MAR mechanism. To evaluate this aim, we compared the performance of 16 SI approaches and 12 MI approaches to fit the REEM tree algorithm and LMM when assessing the association between DBP/SBP and predictor variables such as age, gender, and BMI. We also focused on the R-packages and provided R code for data modeling after using the SI and MI approaches, as well as missing longitudinal data simulation.

The real and simulated data results suggest that the REEM tree algorithm could perform better than parametric longitudinal models. Tree algorithms have some advantages compared to parametric longitudinal models, and we propose that researchers use these methods for future longitudinal studies. These algorithms can accommodate large data sets, non-linear relationships, and interactions, and can extract homogeneous subgroups of data. The interpretation of the tree algorithm is straightforward because the result is graphically shown and is robust to multicollinearity and outliers. These algorithms are also invariant to monotone transformations of independent variables and do not require additional distributional assumptions [67, 68, 72–75].

Generally, the comparisons of imputation methods indicated little difference between them. However, a SI approach (traj-mean) had better performance among all imputation approaches in fitting the REEM tree algorithm for both outcome variables DBP and SBP.

In addition, we evaluated the computational time of imputation approaches. JM approaches are much more resource-intensive than FCS approaches. These methods may not be practicable in longitudinal studies with many clusters and predictor variables with high missing rates. In FCS approaches, the convergence of estimations can occur with 5 or 10 iterations. The number of iterations to establish convergence of JM approaches is much larger than FCS approaches, and FCS approaches have higher computational speed than SI approaches. Overall, MI approaches require more computing resources than SI approaches. Therefore, in real applications with many clusters (e.g., TCGS), SI approaches are likely to be more cost-effective in terms of computational time. The SI approaches are not based on the maximum likelihood estimation and are classified as non-parametric imputation approaches. These methods are appropriate for non-parametric trajectories (e.g., BMI trajectories in TCGS data). Based on the computational time and performance of SI method of traj-mean, it appears this can be an excellent approach to deal with missing observations in data similar to the TCGS data set.

In the current study, we only assessed the MCAR mechanism, though there are sensitivity analyses (selection models and pattern-mixture models) that can be performed to assess the appropriateness of the MAR assumption; unfortunately, these models are unavailable for longitudinal data with missing values in longitudinal quantitative predictor and outcome variables [17, 76–78].

Past studies compared MI approaches for fitting parametric longitudinal models, such as LMM with random intercepts and LMM with random intercepts and slopes. These studies indicate that all MI approaches provide consistent regression coefficients [16, 17]. Some studies also compared these imputation methods for missing data in the context of multilevel data and concluded that these methods provide consistent regression coefficients [15]. In addition, two studies comprehensively compared the SI approaches to impute monotone and non-monotone missing data in longitudinal studies. Unlike the current study, the copy mean method was more effective than other SI approaches [34. Like these two studies, Zhang (2016) also indicated that the copy-mean method had better performance for imputing missing values [79].

To the best of our knowledge, the present study is the first to compare the SI, and MI approaches to impute missing longitudinal data with many time points, clusters, and values using real and simulation data. However, this present study has two limitations, one of which was related to the violation of parametric longitudinal model assumptions. Another limitation was related to the computational time of the simulation study. We used SI approaches and a non-parametric longitudinal model like the REEM tree algorithm to deal with this limitation. For future studies, the non-parametric imputation methods using multivariate skew-normal distribution for the random effects can impute missing longitudinal data. In addition, in the case with unequal time intervals, functional data analysis could be helpful.

## Conclusion

The result of this study should be generalized with caution to other data sets with different characteristics. Because imputation methods can have different levels of performance with different data sets, certain conditions such as missing the data mechanisms or the rate of missingness might lead analysts to opt for different imputation options. Therefore, we conclude that researchers apply all imputation methods (SI and MI) in the context of fitting their statistical models, and then select the imputation method that demonstrates the best performance based on the criteria highlighted in this paper.

## Supplementary Information

**Additional file 1:** **Figure S1.** The process of the multiple imputations approach (e.g., the number ofmultiple imputed data sets is equal to 5).

**Additional file 2:** **Figure S2.** Missing data pattern (percentage of missing values in a particular combination of variables based on the long data format) using VIM package (blue color: observed values andred color: missing values).

**Additional file 3:** **Figure S3.** The plot of standardized residuals versus fitted values for linear mixed-effects model with random intercepts using lme4 package based on the model: DBP ~ Age + Sex + BMI + Time + (1|Id) after using the traj-mean method for the imputation of missing values of longitudinal data.

**Additional file 4:** **Figure S4.** The plot of standardized residuals versus fitted values for linear mixed-effects model with random intercepts using lme4 package based on the model: SBP ~ Age + Sex + BMI + Time + (1|Id) after using the traj-mean method for the imputation of missing values of longitudinal data.

**Additional file 5:** **Figure S5.** The quantile-quantile plot of residuals for linear mixed-effects model with random intercepts using lme4 package based on the model: DBP ~ Age + Sex + BMI + Time + (1|Id) after using the traj-mean method for the imputation of missing values of longitudinal data.

**Additional file 6:** **Figure S6.** The quantile-quantile plot of residuals for linear mixed-effects model with random intercepts using lme4 package based on the model: SBP ~ Age + Sex + BMI + Time + (1|Id) after using the traj-mean method for the imputation of missing values of longitudinal data.

**Additional file 7:** **Figure S7.** Standardized residuals of linear mixed-effects model with random intercepts versus BMI variable using lme4 package based on the model: DBP ~ Age + Sex+ BMI + Time + (1|Id) after using the traj-mean method for the imputation of missing values of longitudinal data.

**Additional file 8:** **Figure S8.** Standardized residuals oflinear mixed-effects model with random intercepts versus BMI variable using lme4 package based on the model: SBP ~ Age + Sex+ BMI + Time + (1|Id) after using the traj-mean method for the imputation of missing values of longitudinal data.

**Additional file 9:** **Figure S9.** The tree structure of the REEMtree algorithm based on the traj-mean method to impute missing values for extracting homogeneous subgroups ofobservations for diastolic blood pressure (DBP) using the REEMtree package.This tree algorithm extracted 7 homogeneous subgroups of observations; the lowest and highest subgroups were subjects with "BMI < 23.70 & age< 41.50" and subjects with "BMI ≥ 30.41 & age ≥ 43.50", respectively.

**Additional file 10:** **Figure S10.** The tree structure of the REEMtree algorithm based on the traj-mean method to impute missing values for extracting homogeneous subgroups ofobservations for systolic blood pressure (SBP) using the REEMtree package. This tree algorithm extracted 9 homogeneous subgroups of observations; the lowest and highest subgroups were subjects with "BMI < 23.70 & age <49.50" and subjects with "age ≥ 59.50 & BMI ≥ 26.50", respectively.

**Additional file 11:** **Figure S11.** Density plots of the observed and imputed data for incomplete variables like BMI and DBP for each iteration (the number of iterations = 20) using mice package (observed data: blue and imputed data: red).

**Additional file 12:** **Figure S12.** Density plots of the observed and imputed data for BMI variablefor each iteration using mice package (observed data: blue and imputed data:red).

**Additional file 13:** **Figure S13.** Density plots of the observed and imputed data for the DBP variable for each iteration using mice package(observed data: blue and imputed data: red).

**Additional file 14:** **Figure S14.** Density plots of the observed and imputed data for the SBP variable for each iteration using mice package (observed data: blue and imputeddata: red).

**Additional file 15:** **Figure S15.** The trace line plots of the mean and standard deviation of the imputed values against the iteration number for each replication using the mice package.

**Additional file 16:** **Figure S16.** Trace plot using JointAI package based on the model: DBP ~ Age + Sex + BMI + Time +(1|Id).

**Additional file 17:** **Figure S17.** MC plot using JointAI package based on the model: DBP ~ Age + Sex + BMI + Time +(1|Id).

**Additional file 18:** **Figure S18.** Trace plot using JointAI package based on the model: SBP ~ Age + Sex + BMI + Time +(1|Id).

**Additional file 19:** **Figure S19.**MC plot using JointAI package based on the model: SBP ~ Age + Sex + BMI + Time + (1|Id).

**Additional file 20:** **Figure S20**. The convergence plot of the JM-MLMM method.

**Additional file 21:** **TableS1.** The sample data for the first 20 individuals of TCGS (NA: missing value).

**Additional file 22:** **Table S2.** Descriptive statistics for continuous variables of TCGS participants included inthe present study at each phase (BMI: body mass index, DBP:diastolic blood pressure, SBP: systolic blood pressure, and SD: std. deviation).

**Additional file 23:** **Table S3.** The statistic for mean and variances for all incomplete variables using miceadds package for FCS-LMM-LN and FCS-LMM-LN-het imputation methods.

## Data Availability

The datasets analyzed during the current study are not publicly available due to containing information that could compromise the privacy of research participants but are available from the corresponding authors on reasonable request. However, R code of SI and MI approaches for missing data imputation, and data simulation are available in https://github.com/MinaJahangiri/R-codes-of-missing-imputation-methods.

## Abbreviations

DBP: Diastolic blood pressure
SBP: Systolic blood pressure
BMI: Body mass index
JM: Joint modelling
FCS: Fully conditional specification
JM-MVN: Joint multivariate normal imputation
SI: Single imputation
MI: Multiple imputation
LMM: Linear mixed-effects model
REEM: Random effects expectation–maximization
TCGS: Tehran cardiometabolic genetic study
