# Supplementary material for: A wide range of missing imputation approaches in longitudinal data: a simulation study and real data analysis
Source: BMC Med Res Methodol. 2023 Jul 6;23:161. doi: 10.1186/s12874-023-01968-8 (PMC10327316; doi:10.1186/s12874-023-01968-8)
Supplement: Supplementary file 1 — Additional file 1: Figure S1. The process of the multiple imputations approach (e.g., the number ofmultiple imputed data sets is equal to 5). [file 12874_2023_1968_MOESM1_ESM.docx]

Dataset with missing values

Final results

Imputation step

Analysis step

Figure S1. The process of the multiple imputations approach (e.g., the number of multiple imputed data sets is equal to 5)
