# Supplementary material for: A wide range of missing imputation approaches in longitudinal data: a simulation study and real data analysis
Source: BMC Med Res Methodol. 2023 Jul 6;23:161. doi: 10.1186/s12874-023-01968-8 (PMC10327316; doi:10.1186/s12874-023-01968-8)
Supplement: Supplementary file 2 — Additional file 2: Figure S2. Missing data pattern (percentage of missing values in a particular combination of variables based on the long data format) using VIM package (blue color: observed values andred color: missing values). [file 12874_2023_1968_MOESM2_ESM.docx]

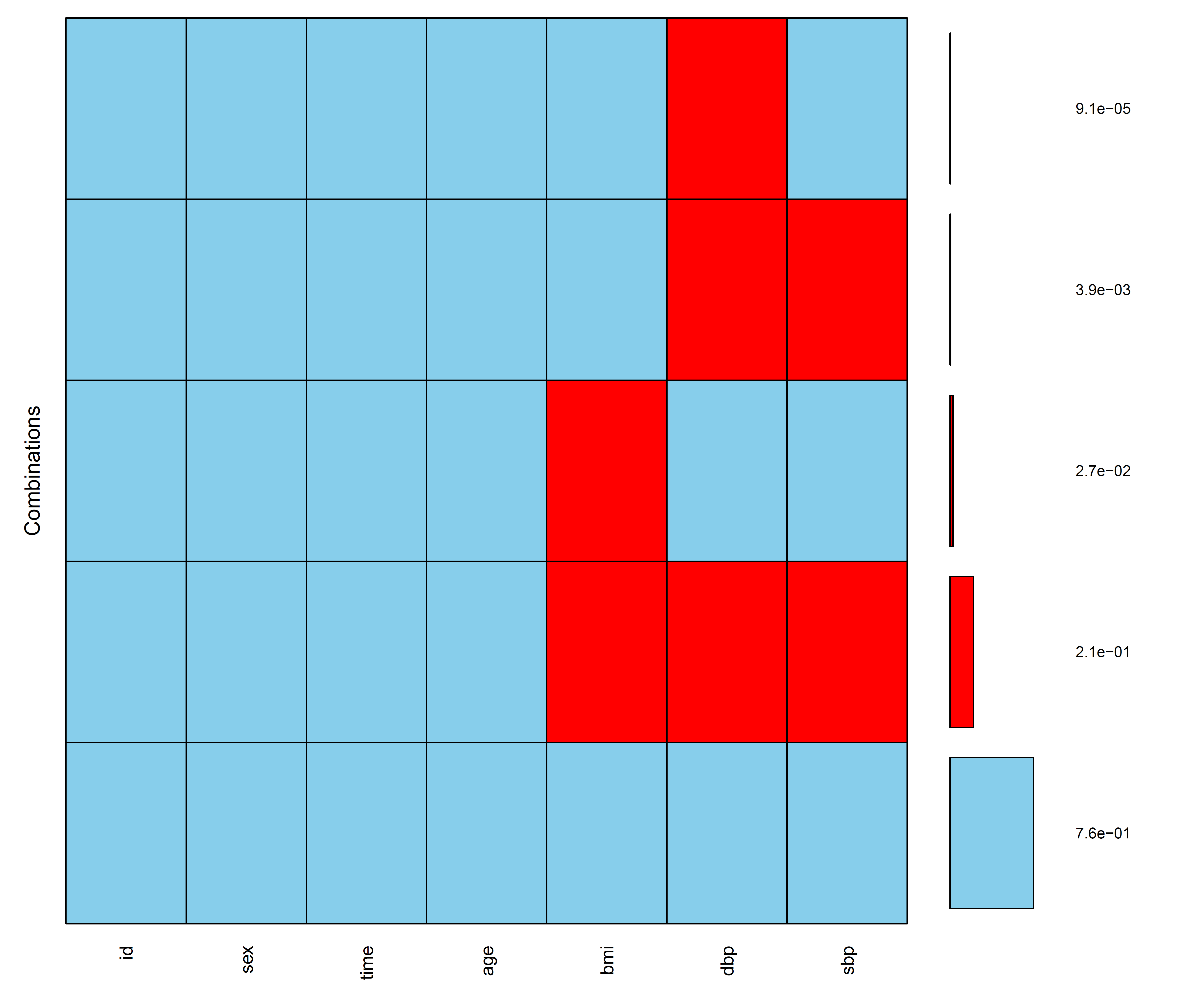


Figure S2. Missing data pattern (percentage of missing values in a particular combination of variables based on the long data format) using VIM package (blue color: observed values and red color: missing values).
