# Supplementary material for: A wide range of missing imputation approaches in longitudinal data: a simulation study and real data analysis
Source: BMC Med Res Methodol. 2023 Jul 6;23:161. doi: 10.1186/s12874-023-01968-8 (PMC10327316; doi:10.1186/s12874-023-01968-8)
Supplement: Supplementary file 7 — Additional file 7: Figure S7. Standardized residuals of linear mixed-effects model with random intercepts versus BMI variable using lme4 package based on the model: DBP ~ Age + Sex+ BMI + Time + (1|Id) after using the traj-mean method for the imputation of missing values of longitudinal data. [file 12874_2023_1968_MOESM7_ESM.docx]

Figure S7. Standardized residuals of linear mixed-effects model with random intercepts on BMI variable using lme4 package based on the model: DBP ~ Age + Sex + BMI + Time + (1|Id) after using the traj-mean method for the imputation of missing values of longitudinal data.
