# Supplementary material for: A wide range of missing imputation approaches in longitudinal data: a simulation study and real data analysis
Source: BMC Med Res Methodol. 2023 Jul 6;23:161. doi: 10.1186/s12874-023-01968-8 (PMC10327316; doi:10.1186/s12874-023-01968-8)
Supplement: Supplementary file 9 — Additional file 9: Figure S9. The tree structure of the REEMtree algorithm based on the traj-mean method to impute missing values for extracting homogeneous subgroups ofobservations for diastolic blood pressure (DBP) using the REEMtree package.This tree algorithm extracted 7 homogeneous subgroups of observations; the lowest and highest subgroups were subjects with "BMI < 23.70 & age< 41.50" and subjects with "BMI ≥ 30.41 & age ≥ 43.50", respectively. [file 12874_2023_1968_MOESM9_ESM.docx]

**70.86**

**73.74**

**79.13**

**79.26**

**75.85**

**75.86**

**82.01**

Figure S9. The tree structure of the REEM tree algorithm based on the traj mean method to impute missing values for extracting homogeneous subgroups of observations for diastolic blood pressure (DBP) using the REEMtree package. This tree algorithm extracted 7 homogeneous subgroups of observations; the lowest and highest subgroups were subjects with "BMI < 23.70 & age < 41.50" and subjects with "BMI ≥ 30.41 & age ≥ 43.50", respectively.
