# Supplementary material for: A wide range of missing imputation approaches in longitudinal data: a simulation study and real data analysis
Source: BMC Med Res Methodol. 2023 Jul 6;23:161. doi: 10.1186/s12874-023-01968-8 (PMC10327316; doi:10.1186/s12874-023-01968-8)
Supplement: Supplementary file 11 — Additional file 11: Figure S11. Density plots of the observed and imputed data for incomplete variables like BMI and DBP for each iteration (the number of iterations = 20) using mice package (observed data: blue and imputed data: red). [file 12874_2023_1968_MOESM11_ESM.docx]

Figure S11. Density plots of the observed and imputed data for incomplete variables like BMI and DBP for each iteration (the number of iterations = 20) using mice package (observed data: blue and imputed data: red).
