# Supplementary material for: A wide range of missing imputation approaches in longitudinal data: a simulation study and real data analysis
Source: BMC Med Res Methodol. 2023 Jul 6;23:161. doi: 10.1186/s12874-023-01968-8 (PMC10327316; doi:10.1186/s12874-023-01968-8)
Supplement: Supplementary file 12 — Additional file 12: Figure S12. Density plots of the observed and imputed data for BMI variablefor each iteration using mice package (observed data: blue and imputed data:red). [file 12874_2023_1968_MOESM12_ESM.docx]

Figure S12. Density plots of the observed and imputed data for BMI variable for each iteration using mice package (observed data: blue and imputed data: red)
