# Supplementary material for: A wide range of missing imputation approaches in longitudinal data: a simulation study and real data analysis
Source: BMC Med Res Methodol. 2023 Jul 6;23:161. doi: 10.1186/s12874-023-01968-8 (PMC10327316; doi:10.1186/s12874-023-01968-8)
Supplement: Supplementary file 15 — Additional file 15: Figure S15. The trace line plots of the mean and standard deviation of the imputed values against the iteration number for each replication using the mice package. [file 12874_2023_1968_MOESM15_ESM.docx]

Figure S15. The trace line plots of the mean and standard deviation of the imputed values against the iteration number for each replication using the mice package
