# Supplementary material for: A wide range of missing imputation approaches in longitudinal data: a simulation study and real data analysis
Source: BMC Med Res Methodol. 2023 Jul 6;23:161. doi: 10.1186/s12874-023-01968-8 (PMC10327316; doi:10.1186/s12874-023-01968-8)
Supplement: Supplementary file 16 — Additional file 16: Figure S16. Trace plot using JointAI package based on the model: DBP ~ Age + Sex + BMI + Time +(1|Id). [file 12874_2023_1968_MOESM16_ESM.docx]

Figure S16. Trace plot using JointAI package based on the model: DBP ~ Age + Sex + BMI + Time + (1|Id)
