# Supplementary figures and images for: A wide range of missing imputation approaches in longitudinal data: a simulation study and real data analysis
Source: BMC Med Res Methodol. 2023 Jul 6;23:161. doi: 10.1186/s12874-023-01968-8 (PMC10327316; doi:10.1186/s12874-023-01968-8)

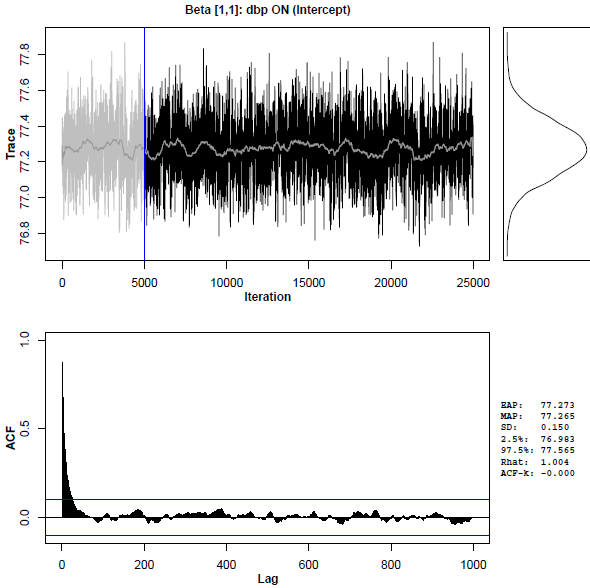


Figure S20. The convergence plot of the JM-MLMM method

Supplement: Supplementary file 20 — Additional file 20: Figure S20. The convergence plot of the JM-MLMM method. [file 12874_2023_1968_MOESM20_ESM.docx]
