# Supplementary material for: A wide range of missing imputation approaches in longitudinal data: a simulation study and real data analysis
Source: BMC Med Res Methodol. 2023 Jul 6;23:161. doi: 10.1186/s12874-023-01968-8 (PMC10327316; doi:10.1186/s12874-023-01968-8)
Supplement: Supplementary file 21 — Additional file 21: TableS1. The sample data for the first 20 individuals of TCGS (NA: missing value). [file 12874_2023_1968_MOESM21_ESM.docx]

| Sex | bmi1 | bmi2 | bmi3 | bmi4 | bmi5 | bmi6 | dbp1 | dbp2 | dbp3 | dbp4 | dbp5 | dbp6 | sbp1 | sbp2 | sbp3 | sbp4 | sbp5 | sbp6 | age1 | age2 | age3 | age4 | age5 | age6 |
| --- | --- | --- | --- | --- | --- | --- | --- | --- | --- | --- | --- | --- | --- | --- | --- | --- | --- | --- | --- | --- | --- | --- | --- | --- |
| Male | NA | 24.77 | 24.73 | 23.72 | 25.14 | 24.4898 | NA | 71 | 79 | 77 | 87 | 76 | NA | 115 | 100 | 113 | 123 | 109 | NA | 42 | 45 | 48 | 51 | 54 |
| Female | NA | 28.25 | 28.67 | 29.05 | 28.67 | 29.55255 | NA | 64 | 65 | 69 | 75 | 74 | NA | 99 | 105 | 98 | 106 | 108 | NA | 36 | 39 | 42 | 45 | 48 |
| Male | NA | 22.89 | 22.84 | 25.03 | 26.86 | 25.35926 | NA | 75 | 76 | 65 | 70 | 69 | NA | 120 | 110 | 97 | 95 | 90 | NA | 25 | 28 | 31 | 34 | 37 |
| Female | 18.34 | 19.96 | 19.96 | NA | 19.72 | 23.45856 | 77 | 70 | 80 | 72 | 70 | 71 | 104 | 95 | 90 | 101 | 92 | 91 | 17 | 20 | 23 | 26 | 29 | 32 |
| Female | 20.66 | 20.81 | 23.31 | 25.39 | 24.97 | 22.89282 | 80 | 80 | 60 | 75 | 70 | 62 | 110 | 104 | 82 | 106 | 96 | 103 | 14 | 17 | 20 | 23 | 26 | 29 |
| Male | 30.42 | 31.12 | 32.11 | 33.79 | 34.14 | 35.75128 | 82 | 80 | 79 | 80 | 80 | 105 | 122 | 112 | 119 | 115 | 113 | 148 | 31 | 34 | 37 | 40 | 43 | 46 |
| Female | 43.51 | 46.47 | 48.65 | 51.12 | 53.42 | NA | NA | 94 | 120 | 103 | 112 | NA | NA | 159 | 150 | 150 | 156 | NA | 52 | 55 | 58 | 61 | 64 | 67 |
| Female | NA | 26.5 | 27.64 | 23.73 | 26.5 | 28.47989 | NA | 44 | 80 | 75 | 59 | 52 | NA | 100 | 110 | 96 | 107 | 105 | NA | 14 | 17 | 20 | 23 | 26 |
| Male | NA | 26.78 | NA | 27.1 | 27.12 | 28.40816 | NA | 67 | NA | 73 | 65 | 71 | NA | 101 | NA | 105 | 90 | 107 | NA | 33 | 36 | 39 | 42 | 45 |
| Male | NA | 24.74 | NA | 24.68 | 25.1 | 25.5102 | NA | 74 | NA | 85 | 71 | 78 | NA | 115 | NA | 140 | 122 | 160 | NA | 58 | 61 | 64 | 67 | 70 |
| Female | NA | 29.15 | 28.31 | 31.47 | 31.93 | 31.46837 | NA | 64 | 80 | 80 | 81 | 72 | NA | 105 | 123 | 119 | 111 | 134 | NA | 30 | 33 | 36 | 39 | 42 |
| Female | NA | 25.22 | 25.28 | 23.71 | 25.97 | 24.38653 | NA | 85 | 87 | 78 | 82 | 77 | NA | 132 | 138 | 120 | 126 | 130 | NA | 58 | 61 | 64 | 67 | 70 |
| Male | NA | 29.76 | 31.14 | 31.44 | 32.15 | 34.54054 | NA | 70 | 70 | 76 | 71 | 77 | NA | 97 | 99 | 95 | 98 | 108 | NA | 24 | 27 | 30 | 33 | 36 |
| Female | NA | 33.78 | 30.96 | 33.78 | 31.39 | 34.72057 | NA | 62 | 58 | 74 | 60 | 80 | NA | 100 | 89 | 100 | 80 | 105 | NA | 42 | 45 | 48 | 51 | 54 |
| Male | 31.62 | 32.36 | 33.18 | 34.25 | NA | NA | 104 | 91 | 99 | 87 | NA | NA | 154 | 137 | 155 | 140 | NA | NA | 65 | 68 | 71 | 74 | 77 | 80 |
| Male | 22.21 | 24.46 | 25.22 | 25.59 | 19.489 | 26.44628 | 67 | 64 | 67 | 74 | 72 | 73 | 111 | 102 | 106 | 112 | 112 | 105 | 23 | 26 | 29 | 32 | 35 | 38 |
| Female | NA | NA | NA | NA | NA | 22.06035 | NA | 57 | 61 | 70 | 70 | 70 | NA | 114 | 110 | 100 | 100 | 110 | NA | 20 | 23 | 26 | 29 | 32 |
| Male | NA | 31.98 | NA | NA | 28.6 | 29.3724 | NA | 95 | NA | NA | 80 | 79 | NA | 144 | NA | NA | 142 | 120 | NA | 60 | 63 | 66 | 69 | 72 |
| Female | NA | 26.04 | NA | NA | 29.64 | NA | NA | 61 | NA | NA | 76 | NA | NA | 92 | NA | NA | 99 | NA | NA | 20 | 23 | 26 | 29 | 32 |

Table S1. The sample data for the first 20 individuals of TCGS (NA: missing value)
