# Supplementary material for: A wide range of missing imputation approaches in longitudinal data: a simulation study and real data analysis
Source: BMC Med Res Methodol. 2023 Jul 6;23:161. doi: 10.1186/s12874-023-01968-8 (PMC10327316; doi:10.1186/s12874-023-01968-8)
Supplement: Supplementary file 22 — Additional file 22: Table S2. Descriptive statistics for continuous variables of TCGS participants included inthe present study at each phase (BMI: body mass index, DBP:diastolic blood pressure, SBP: systolic blood pressure, and SD: std. deviation). [file 12874_2023_1968_MOESM22_ESM.docx]

Table S2. Descriptive statistics for continuous variables of TCGS participants included in the present study at each phase (BMI: body mass index, DBP: diastolic blood pressure, SBP: systolic blood pressure, and SD: std. deviation)

| Variable | Descriptive statistics | Follow up 1 | Follow up 2 | Follow up 3 | Follow up 4 | Follow up 5 | Follow up 6 |
| --- | --- | --- | --- | --- | --- | --- | --- |
| Age | Mean | 39.34 | 42.68 | 45.75 | 49.12 | 52.35 | 55.56 |
|  | SD | 16.23 | 16.25 | 16.17 | 16.19 | 16.15 | 16.14 |
| BMI | Mean | 26.36 | 27.29 | 27.59 | 28.21 | 28.44 | 28.55 |
|  | SD | 5.19 | 4.95 | 4.86 | 4.87 | 5.01 | 4.85 |
| DBP | Mean | 77.87 | 75.42 | 74.48 | 78.44 | 79.32 | 78.29 |
|  | SD | 11.08 | 11.13 | 11.12 | 11.72 | 11.21 | 10.94 |
| SBP | Mean | 118.94 | 117.67 | 116.43 | 119.87 | 121.37 | 120.34 |
|  | SD | 19.28 | 19.67 | 19.99 | 19.89 | 20.43 | 19.82 |
