# Supplementary material for: A wide range of missing imputation approaches in longitudinal data: a simulation study and real data analysis
Source: BMC Med Res Methodol. 2023 Jul 6;23:161. doi: 10.1186/s12874-023-01968-8 (PMC10327316; doi:10.1186/s12874-023-01968-8)
Supplement: Supplementary file 23 — Additional file 23: Table S3. The statistic for mean and variances for all incomplete variables using miceadds package for FCS-LMM-LN and FCS-LMM-LN-het imputation methods. [file 12874_2023_1968_MOESM23_ESM.docx]

Table S3. The statistic for mean and variances for all incomplete variables using miceadds package for FCS-LMM-LN and FCS-LMM-LN-het imputation methods

| Incomplete variable | Imputation method | Missing proportion | Rhat.Mean | |
| --- | --- | --- | --- | --- |
|  |  |  | Mean | Variance |
| BMI | FCS-LMM-LN | 23.923 | 0.998 | 0.995 |
|  | FCS-LMM-LN-het |  | 1.0003 | 0.999 |
| DBP | FCS-LMM-LN | 21.664 | 1.007 | 0.985 |
|  | FCS-LMM-LN-het |  | 1.0008 | 0.999 |
| SBP | FCS-LMM-LN | 21.655 | 1.024 | 0.999 |
|  | FCS-LMM-LN-het |  | 1.018 | 1.026 |
